# Nontoxic mesoporous silica nanoparticles protect *Physcomitrium patens* against salt stress

**DOI:** 10.1007/s44154-025-00262-5

**Published:** 2025-11-14

**Authors:** Ying Zhou, Zhuo Yang, Jiaxue Li, Xuemei Xia, Wei Yuan, Chen Li, Wenxiu Qiu, Li Liu, Liu Duan

**Affiliations:** 1 College of Life Science and Health, Wuhan University of Science and Technology, Wuhan, 430065 China; 2 School of Life Sciences, Hubei University, Wuhan, 430062 China; 3 Shiyan Key Laboratory of Medicinal Plants and Evolutionary Genetics, Hubei Key Laboratory of Embryonic Stem Cell Research, School of Basic Medicine, Hubei University of Medicine, Shiyan, 442000 China

**Keywords:** Mesoporous silica nanoparticles (MSNs), Salt stress, *Physcomitrium patens*, Ion homeostasis, ROS homeostasis, Calcium ion signaling

## Abstract

Mosses play a crucial role in environmental protection, ecological preservation, and horticulture. While the effects of nanomaterials on angiosperms have been widely studied, their impact on bryophytes remains underexplored. In this study, we investigated the effects of mesoporous silica nanoparticles (MSNs) and virus-like mesoporous silica nanoparticles (VMSNs) on the model moss species *Physcomitrium patens* (*P. patens*). Our results revealed that MSNs, with an average size of approximately 123 nm, are nontoxic to *P. patens* and enhance its salt tolerance. The expression of key genes involved in stress responses were significantly induced in MSN-treated plants under salt stress, including *peroxidase* (*POX*), *L-ascorbate oxidase* (*L-AO*), *alternative oxidase* (*AOX*), and *calcium-dependent protein kinase* (*CPK*). MSN treatment reduced the accumulation of H_2_O_2_ and O_2_^·−^, increased Ca^2+^ signaling, and modulated reactive oxygen species (ROS) homeostasis, collectively improving moss tolerance to salt stress. MSNs were observed on the cell surface, in intercellular space, and within the cytosol and vesicles. They were transported bidirectionally between rhizoids and apical leaves. This study provides novel insights into the distribution, transport, and functional mechanisms of MSNs in mosses, offering a valuable foundation for the application of nanomaterials in plant stress biology and ecological management of bryophytes.

**Supplementary Information:**

The online version contains supplementary material available at 10.1007/s44154-025-00262-5.

## Introduction

With the challenges posed by global climate change, both the ecological environment and agricultural production are under tremendous pressure (Wang et al. 2024). Soil salinization poses a significant threat to plant growth and development, severely impacting their ability to thrive in environments and affecting the natural geographic distribution of plants. Unlike animals, plants are immobile and must rely on their inherent mechanisms to adapt to harsh conditions. Overtime, plants have evolved various defense strategies to cope with severe environmental challenges (Gong et al. 2020).

Salt stress affects plants at multiple levels, leading to a cascade of responses that impact growth and development. At the cellular level, salt stress often induces lignin accumulation, disrupts pectin cross-linking, and reduces cellulose content, potentially compromising cell wall integrity (Dabravolski and Isayenkov 2023). In *Nicotiana tabacum*, salinized plants exhibit enlargement of upper epidermal cells and a reduction in leaf succulence, as evidenced by decreased leaf water content per unit area, a lower fresh-to-dry weight ratio, and a faster rate of water loss (Han et al. 2013). Physiologically, salt stress significantly impairs photosynthesis. A significant reduction in net photosynthetic rate, maximum photochemical efficiency, or effective quantum yield of PSII has been observed in species such as *Physcomitrium patens* (*P. patens*), tobacco, and Arabidopsis, leading to a decrease in fresh weight per growth area and reduced pigment contents (Han et al. 2013; Xiao et al. 2021; Zhuang et al. 2025). Furthermore, salt stress triggers the production of reactive oxygen species (ROS) within plant cells. While low concentrations of ROS serve as signal molecules, excessive ROS such as superoxide radicals, hydrogen peroxide, and hydroxyl radicals can disrupt cellular functions, impairing plant growth and development. To mitigate ROS induced damage, plants enhance their antioxidant defense systems, including enzymes such as superoxide dismutase (SOD), peroxidase (POX), catalase (CAT), and nonenzymatic antioxidants, which help improve tolerance to salt stress (Singh 2022). Moreover, salt stress disrupts ion homeostasis by affecting the uptake and transport of sodium and potassium ions, as well as maintaining their concentrations (Han et al. 2013; Zhang et al. 2018; Xiao et al. 2021). Calcium signaling also plays a pivotal role in plants response to salt stress, the calcium signaling pathway is highly conserved in the studied terrestrial plants, and its sensor proteins (such as CPKs) are widely involved in stress responses (Manishankar et al. 2018; Xiao et al. 2021). Transcriptomic and proteomic studies have further elucidated the molecular mechanisms underlying salt stress tolerance. Stress-responsive genes, including transcription factors, salt-sensitive genes, ion homeostasis regulators, transporters, and calcium-dependent protein kinases, and stress-response genes, have been identified as potential molecular markers for evaluating salinity stress in plants (Grimplet et al. 2017; Li et al. 2017; Gao et al. 2018; Borkiewicz et al. 2020; Liu et al. 2024). Although plant adaptation to salt stress can be improved by strategies such as developing new salt-tolerant varieties through genetic improvement, choosing and cultivating germplasm with strong salt tolerance, and progress in these areas has been relatively slow and is constrained by numerous factors. Therefore, it remains essential to identify effective and safe methods to enhance plant salt tolerance.

In recent years, nanomaterials have emerged as key players in agriculture, offering innovative solutions to address practical challenges in agricultural production (Zhou et al. 2021; Du et al. 2022). Nano-fertilizers and nano-pesticides, have shown positive effects on plant growth and stress tolerance. For example, ZnO nanoparticles, when applied at optimal concentrations, can improve plant germination and enhance stress tolerance, alleviate toxicity induced by cadmium and aluminum in plants such as pepper and soybean (Kolenčík et al. 2020; Zhang et al. 2024; Tahira et al. 2025). These nanoparticles also increase chlorophyll content and enhance photosynthetic efficiency under environmental stresses. Similarly, SiO₂ nanoparticles improve plant resilience to drought, salt, and cold stress by promoting seed germination, biomass accumulation, and photosynthetic activity (Kalteh et al. 2014; Siddiqui et al. 2014; Bastías et al. 2014; Ashkavand et al. 2016; Du et al. 2022; Tang et al. 2012; Wang et al. 2023). However, the effects of nanoparticles on plant growth are not always beneficial. Several studies have reported adverse impacts, including reduced growth, genotoxicity, cytotoxicity, and oxidative stress. Nanoparticles like ZnO, MgO, MnO and Ag have been shown to induce DNA strand breakage, generate ROS, disrupt chloroplast structure, and impair growth (Ahmed et al. 2017; Liang et al. 2018; Mangalampalli et al. 2018; Ghosh et al. 2019). These dual effects highlight the importance of a careful and comprehensive assessment of nanoparticle applications to ensure their safety and efficacy in agricultural production and environmental protection.

Although significant progress has been made in understanding the impact of nanomaterials on crop plants, studies on their effects in bryophytes remain limited. Mosses, as early land plants, exhibit distinct differences from angiosperms in many respects. Their stress response mechanisms (e.g., calcium signaling pathways) are closer to the origin of plant evolution (Manishankar et al. 2018), providing theoretical insights into nanomaterial-plant interactions. Plants have potential applications in saline soil remediation (Salma et al. 2025), and clarifying how nanomaterials enhance their salt tolerance will guide the design of nanotechnologies for ecological restoration. As a model organism for bryophytes, *P. patens* has a well-established gene editing system, which facilitates in-depth analysis of the molecular mechanisms underlying -enhanced salt tolerance (Rensing et al. 2020). This characteristic makes it an ideal candidate for exploring the functional mechanisms of nanomaterials in plants. They are structurally simple, lacking vascular tissue and true differentiation of roots, stems, and leaves, with their leaves typically consisting of a single layer of cells (Rensing et al. 2020). Despite their structural fragility, mosses exhibit remarkable adaptability to environmental stress and play a critical role in ecosystems. Given their unique physiological and ecological characteristics, mosses are valuable not only as environmental monitoring plants, but also for their ability to restore ecological balance, significantly contributing to soil conservation and water purification (Szczepaniak and Biziuk 2003; Gecheva and Yurukova 2014; Fernández et al. 2015). Although SiO_2_ nanoparticles show potential in improving salt tolerance in higher plants (Rai-Kalal et al. 2021), the transport mechanism, subcellular distribution, and functional association of mesoporous silica nanoparticles (MSNs) in bryophytes (simple early land plants) remain unknown. Specifically, there is a lack of direct evidence on whether MSNs can enhance salt tolerance in mosses. Given the fundamental differences in ion transport and stress response mechanisms between mosses (devoid of vascular tissues) and higher plants (Rensing et al. 2020), conclusions from higher plant studies cannot be extrapolated to mosses. Thus, we highlighted the necessity of exploring MSN mechanism in moss models. In recent years, *P. patens* has garnered increased attention as a model system for study of bryophytes, owing to its extensive genomic resources and advanced functional verification techniques (Rensing et al. 2008, 2020). However, the effects of nanoparticle exposure on *P. patens* remain poorly understood, and the potential impact of moss on broader ecological systems is yet to be fully explored.

**Scheme 1:**
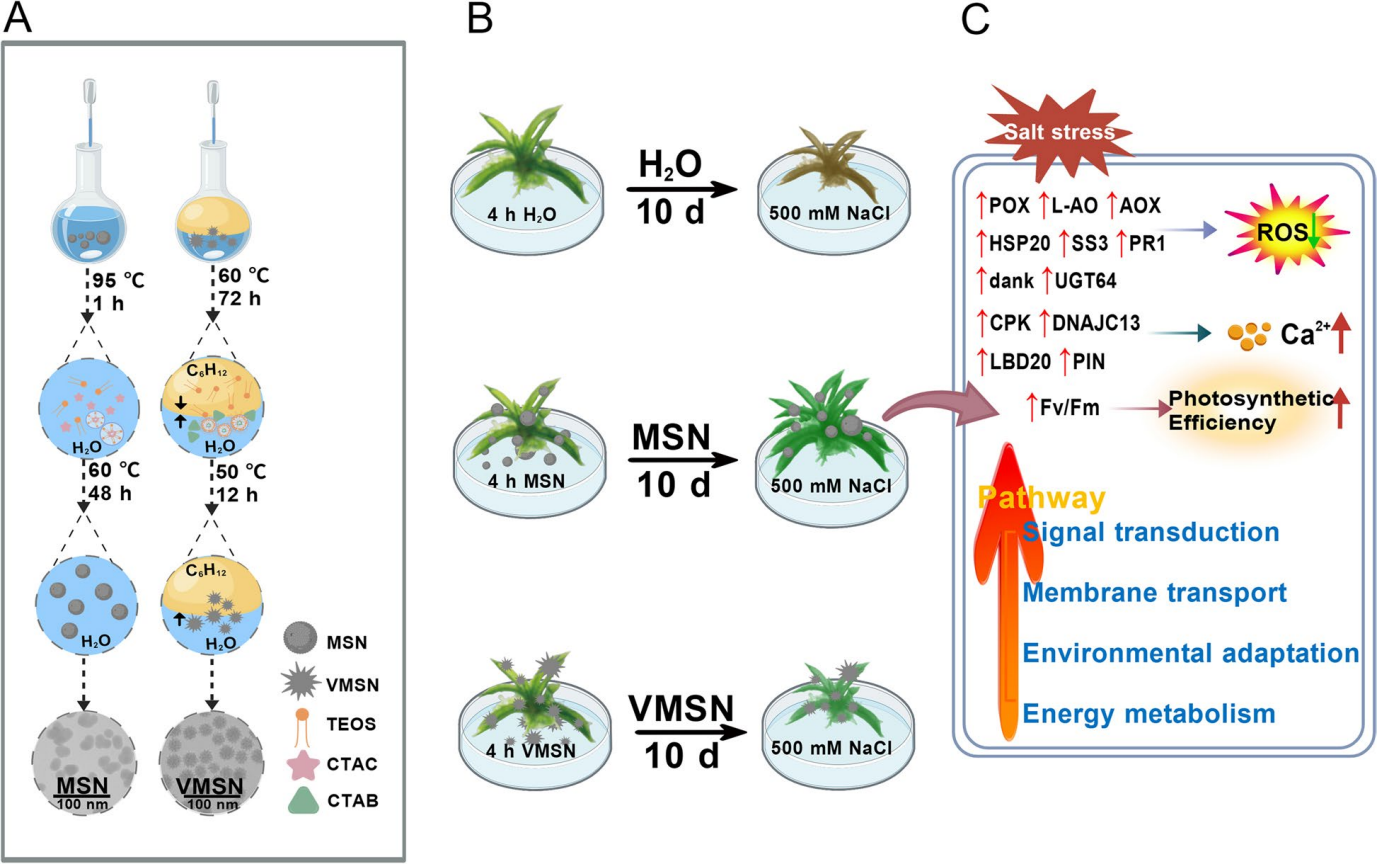
Synthesis and Mechanism of Action of MSN/VMSN in *Physcomitrium patens *(*P. patens*). **A** Synthesis process of MSN and VMSN nanoparticles. **B** Effects of MSN/VMSN treatment on the growth of *P. patens* under salt stress. **C** Expression changes of relevant genes following MSN treatment under salt stress. Created with BioGDP.com

In this study, MSNs were synthesized via an alkaline-catalyzed hydrolysis method, while virus-like mesoporous silica nanoparticles (VMSNs) were obtained using a two-phase diffusion system. The morphology of MSNs and VMSNs was characterized by transmission electron microscopy (TEM), and their size and dispersity were assessed by dynamic light scattering (DLS). Both MSNs and VMSNs were applied to *P. patens* gametophores under NaCl stress. Growth area and survival rates were compared between the nanoparticle-treated and control groups. The maximum quantum yields of photosystem II (PSII), reactive oxygen species levels, and calcium signaling were measured to assess the physiological responses in moss. Fluorescein isothiocyanate labeled MSNs (MSN-FITC), along with TEM observations, were used to investigate the uptake, transport, and distribution of MSNs within *P. patens* cells. RNA sequencing and quantitative reverse transcription PCR (qRT-PCR) were performed to explore the molecular mechanisms underlying. Our results provide new ideas and insights for the application of nanoparticles in plants, as illustrated in Scheme 1.

## Results

### Synthesis and characterization of MSN, VMSN and MSN-FITC

**Fig. 1:**
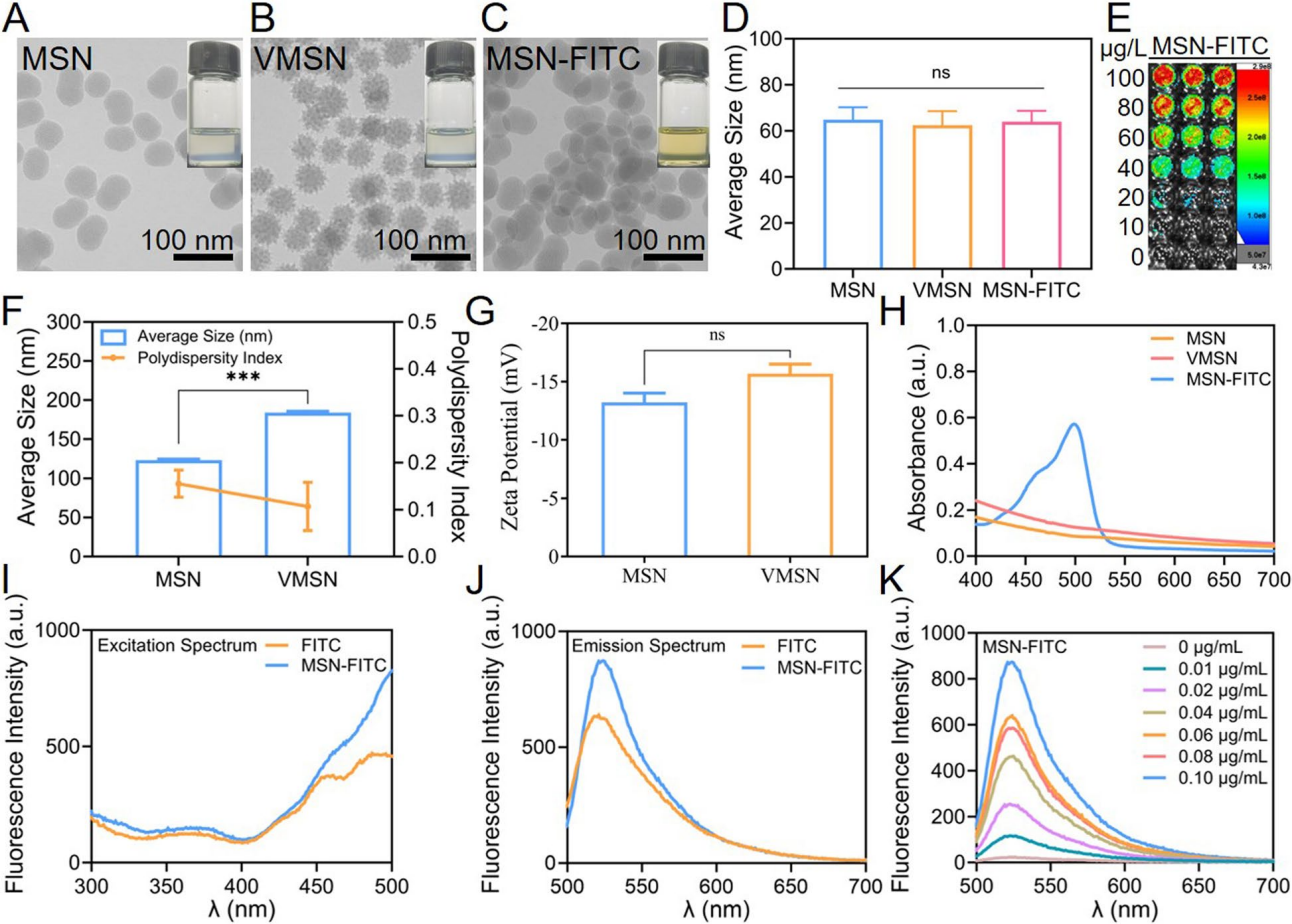
Characterization of MSN, VMSN and MSN-FITC. **A-C** TEM of MSN, VMSN and MSN-FITC. The insets in the top right of each panel show images of nanoparticles in aqueous solution. **D** Average dry-state particle size of MSN, VMSN and MSN-FITC.** E** Fluorescence images of MSN-FITC with different concentration. **F** Average hydrated particle size and polydispersity index (PDI) of MSN and VMSN. **G** Zeta potential measurements of MSN and VMSN in aqueous solution. **H** UV–vis spectra of MSN, VMSN and MSN-FITC in ethanol. **I** Fluorescence excitation spectra and **J** emission spectra of MSNs-FITC and free FITC in ethanol. **K** Fluorescence intensity of MSN-FITC at varying concentrations (Ex = 490 nm, Em = 520 nm). Asterisks indicate the significant difference, ns indicates no significance, *** *p* < 0.001 (*two-way ANOVA*)

In order to understand the effects of two kinds of nano-silica with distinct morphologies on *P. patens*, it was crucial to first confirm the successful synthesis of these nano-silicon structures. TEM imaging revealed that MSN exhibited a porous spherical structure (Fig. 1A), while VMSN displayed a characteristic spiky surface morphology (Fig. 1B). After modification with FITC, the morphological characteristics of MSN remained unchanged, indicating that FITC can be used as a label for tracking MSN within plant cells (Fig. 1C). Additionally, all three types of nano-silica formulations exhibited good dispersity (Fig. 1A and C). Statistical analysis of the TEM results for the three types of nano-silicon revealed that the dry-state particle sizes of MSN, VMSN, and MSN-FITC were 64.84 ± 5.41 nm, 62.49 ± 6.10 nm, and 63.99 ± 4.71 nm, respectively. As shown in Figure S1 and Fig. 1D, there were no significant differences in their dry-state particle sizes.

The hydrated particle size and surface potential of MSN and VMSN nano-silica formulations were evaluated using DLS (Fig. 1F and G). The mean hydrated particle sizes of MSN and VMSN were 123.11 ± 1.39 nm and 184.04 ± 1.54 nm, respectively. The significant difference in their hydrodynamic radii is attributed to their distinct morphologies, which lead to different extents of surface hydration.

Zeta potential measurements showed that both types of nano-silica exhibited negative surface charges. The zeta potential of MSN was −13.23 ± 1.42 mV, and that of VMSN was −15.70 ± 1.51 mV (Fig. 1G), and there were no significant differences in their zeta potential. These results demonstrate that the successful preparation of two nano-silica formulations with distinct morphologies and undifferentiated zeta potential as well as dry-state particle sizes. They are stable in aqueous solution.

Since the subsequent distribution and metabolism of these nano-silica formulations in plants will be assessed using FITC fluorescence, the spectral properties of MSN and MSN-FITC were thoroughly evaluated. Fluorescence imaging of MSN-FITC was first performed using an in vivo imaging system. As shown in Fig. 1E, MSN, after being modified with FITC, exhibited excellent fluorescence imaging properties, with the fluorescence signal intensity increased as the concentration increased. UV–Vis spectroscopy further confirmed the successful modification of FITC onto the surface of MSN, with characteristic absorption peaks observed in the 450–520 nm wavelength range (Fig. 1H).

Fluorescence excitation and emission spectra of MSN-FITC and free FITC revealed that at the same concentration, the fluorescence intensity of MSN-FITC was over 1.5 times higher in excitation and over 1.4 times higher in emission than that of FITC, respectively (Fig. 1I and J). The results confirmed the successful conjugation of FITC to the surface of MSN via chemical synthesis without compromising its fluorescence properties. Notably, MSN-FITC exhibited enhanced fluorescence performance compare to free FITC (Fig. 1I and J), likely due to the hydrophobic nature of FITC and the excellent dispersibility of MSN. This finding suggested that FITC modification can serve as an effective tracing tool of MSN and further highlighted the superior dispersibility of MSN. As shown in Fig. 1K, the fluorescence intensity of MSN-FITC increases with its concentration, indicating that FITC fluorescence can be used for quantitative analysis of nano-silica formulations. These findings are consistent with those presented in Fig. 1E, confirming that MSN showed excellent dispersibility and is an ideal carrier for hydrophobic molecules.

### Enhanced salt tolerance in *P. patens* via MSN and VMSN application

To investigate the effects of MSN and VMSN on the growth or physiological responses of *P. patens* under salt stress conditions, we first examined the plant’s response to various concentrations of NaCl (Figure S2). On day 10 of exposure, protonemata began to emerge in samples treated with lower NaCl concentrations (50 mM and 150 mM), but at a slower rate compared to the control, indicating that these lower concentrations of NaCl exerted a growth-inhibitory effect on *P. patens*. Samples treated with 300 mM NaCl showed no noticeable changes, suggesting that this concentration was sufficient to suppress normal growth. In contrast, samples exposed to 500 mM and 800 mM NaCl exhibited distinct whitish discoloration by day 10, indicating severe damage, with plants turning white and dying at these high concentration (Figure S2). Based on these results, we selected 500 mM NaCl, the lowest concentration causing significant stress, as the concentration for testing the effects of MSNs and VMSNs.

**Fig. 2:**
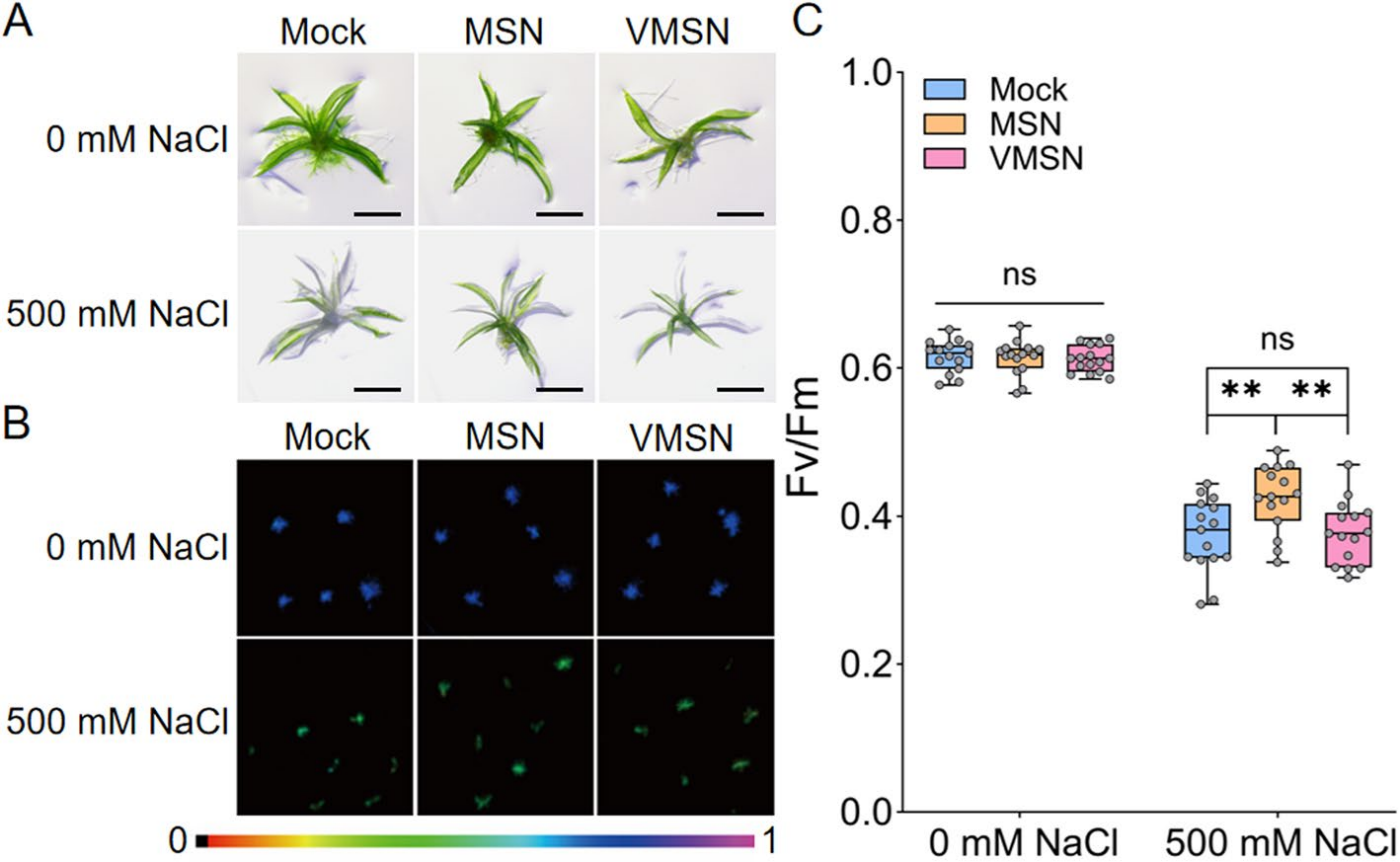
Effects of MSNs and VMSNs on *Physicomitrium patnes* (*P. patens*) under NaCl stress. **A** Representative images of *P. patens* exposed to 500 mM NaCl with MSN or VMSN treatment. Tips (2–3 mm) excised from one-month-old *P. patens* gametophores were soaked in 300 mg/L MSN or VMSN for 4 h and then washed with sterile water. The tips were transferred to BCD medium containing 500 mM NaCl, with 0 mM NaCl as the control. Images were taken on day 10 of treatment. Scale bar = 1 mm. **B** Representative images of chlorophyll fluorescence Fv/Fm parameter of the plants under salt stress for 6 days.** C** Quantification of chlorophyll fluorescence Fv/Fm parameter under stress for 6 days. Data are presented as means ± SEM (*n* = 15). Asterisks indicate the significant difference, ns indicates no significance, ** *p* < 0.01 (*two-way ANOVA*)

Next, different concentrations of MSN and VMSN were applied to assess their effects on *P. patens* growth and salt tolerance. The results showed that, in the absence of salt stress, neither MSN nor VMSN had a significant impact on plant growth or the Maximum quantum yield of photosystem II (Fv/Fm) photosynthetic rate (Fig. 2). After treatment with 500 mM NaCl, plants without the application of nanoparticles exhibited significant chlorosis, with the Fv/Fm value dropping from 0.6158 ± 0.005559 to 0.3737 ± 0.012972 by day 6. In contrast, plants treated with MSNs at concentrations of 50 mg/L, 100 mg/L and 300 mg/L displayed improved tolerance to NaCl toxicity, as evidenced by reduced chlorosis at both 10 and 15 days (Figure S3A), and the Fv/Fm value was 0.4243 ± 0.011606 at 6 days under 300 mg/L MSN treatment, significantly higher than that of the untreated plants (Fig. 2B and C). However, treatment with VMSN, regardless of concentration, showed no substantial improvement over the untreated controls (Figure S3B, Fig. 2B and C). These results suggest that high-concentration MSN treatment can effectively alleviate salt stress induced by 500 mM NaCl in *P. patens*.

**Fig. 3:**
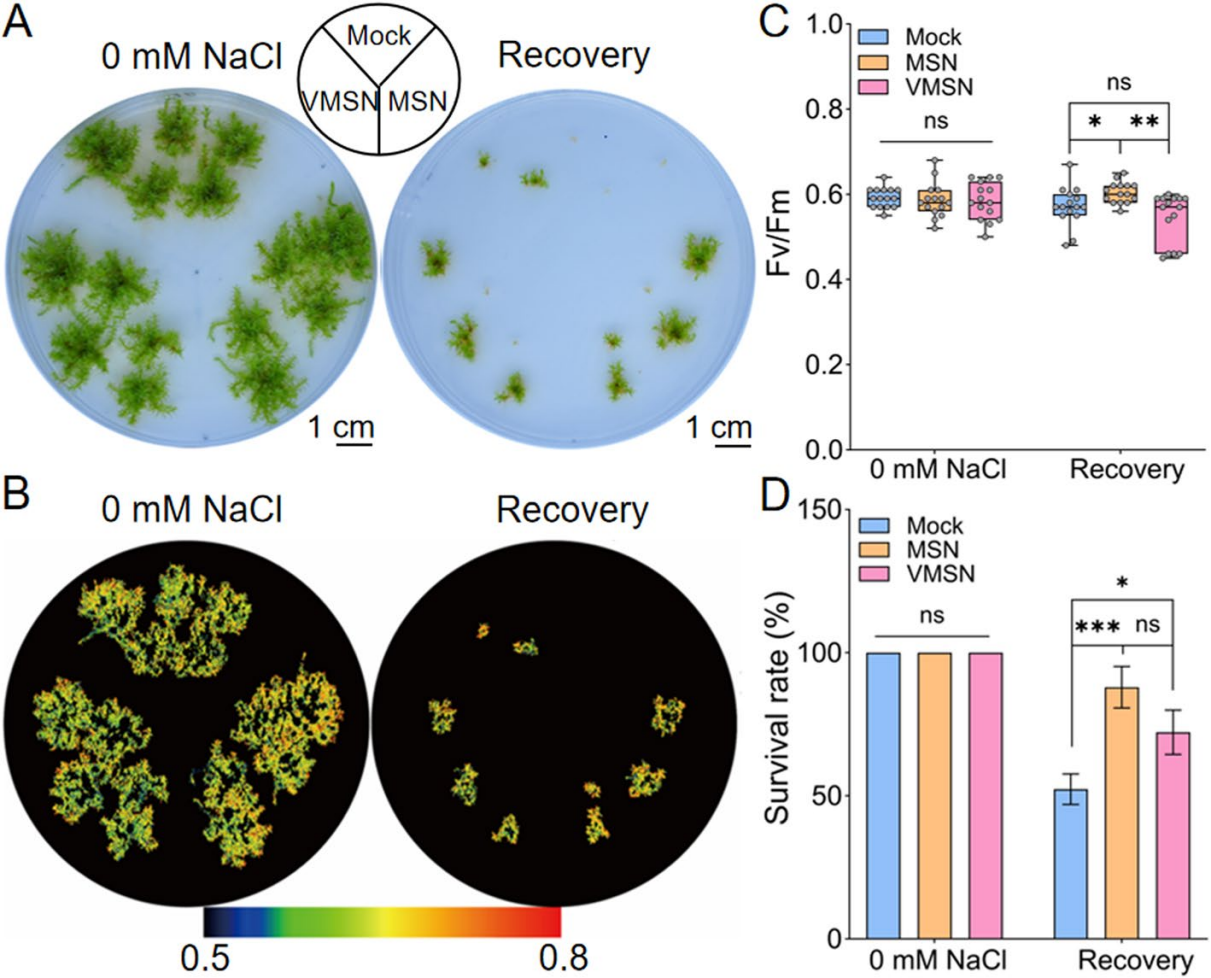
Effects of MSNs and VMSNs on *P. patens* at the recovery stage after salt stress. **A** Representative images of *P. patens* during the recovery stage after exposed to 500 mM NaCl with MSN or VMSN treatment. Tips (2–3 mm) excised from one-month-old *P. patens* gametophores were soaked in 300 mg/L MSN or VMSN for 4 h and washed with sterile water. The tips were first exposed to 500 mM NaCl for 10 days, followed by 60 days of recovery in BCD medium without NaCl. Scale bar = 1 cm. **B** Representative images of chlorophyll fluorescence Fv/Fm during the recovery stage after salt stress.** C** Quantification of chlorophyll fluorescence Fv/Fm during the recovery stage after salt stress. Data are presented as means ± SEM (*n* = 15). Asterisks indicate the significant difference between mock and 300 mg/L MSN or VMSN. **D** Survival rates of *P. patens* during the recovery stage after salt stress. The survival rate is calculated as the ratio of viable gametophytes to total gametophytes per dish in BCD medium. Data from three biological replicates, with each replicate comprising a group of over 8 gametophores, are presented as means ± SEM. Asterisks indicate significant differences, *p < 0.05, **p < 0.01, ***p < 0.001 (*two-way ANOVA*, *Tukey test*), ns indicates no significance

To further investigate the effects of MSNs and VMSNs on the recovery of *P. patens* after the removal of NaCl stress, we compared the recovery process of plants placed on BCD medium for 60 days following a 10-day exposure to 500 mM NaCl (Fig. 3). The results showed that, after experiencing NaCl stress, only 52.30% of the plants were able to survive (Fig. 3A and D) and regain their photosynthetic capacity (Fig. 3B and C). In contrast, following MSN and VMSN treatment, the survival rates after recovery increased to 87.96% and 72.22%, respectively, representing a 1.68 and 1.38 fold improvement compared to the control group without nanoparticle treatment. Meanwhile, the growth area of the surviving plants was 36.40% of that observed in non-stressed plants, and there was no significant difference in the growth area of the surviving plants (Figure S4). Notably, MSN treatment significantly enhanced the Fv/Fm of the plants after stress relief (Fig. 3C) and promoted the recovery of *P. patens*, further indicating the beneficial role of MSN in the recovery process.

### MSN alleviates salt stress by modulating ROS accumulation and affecting calcium signaling

**Fig. 4:**
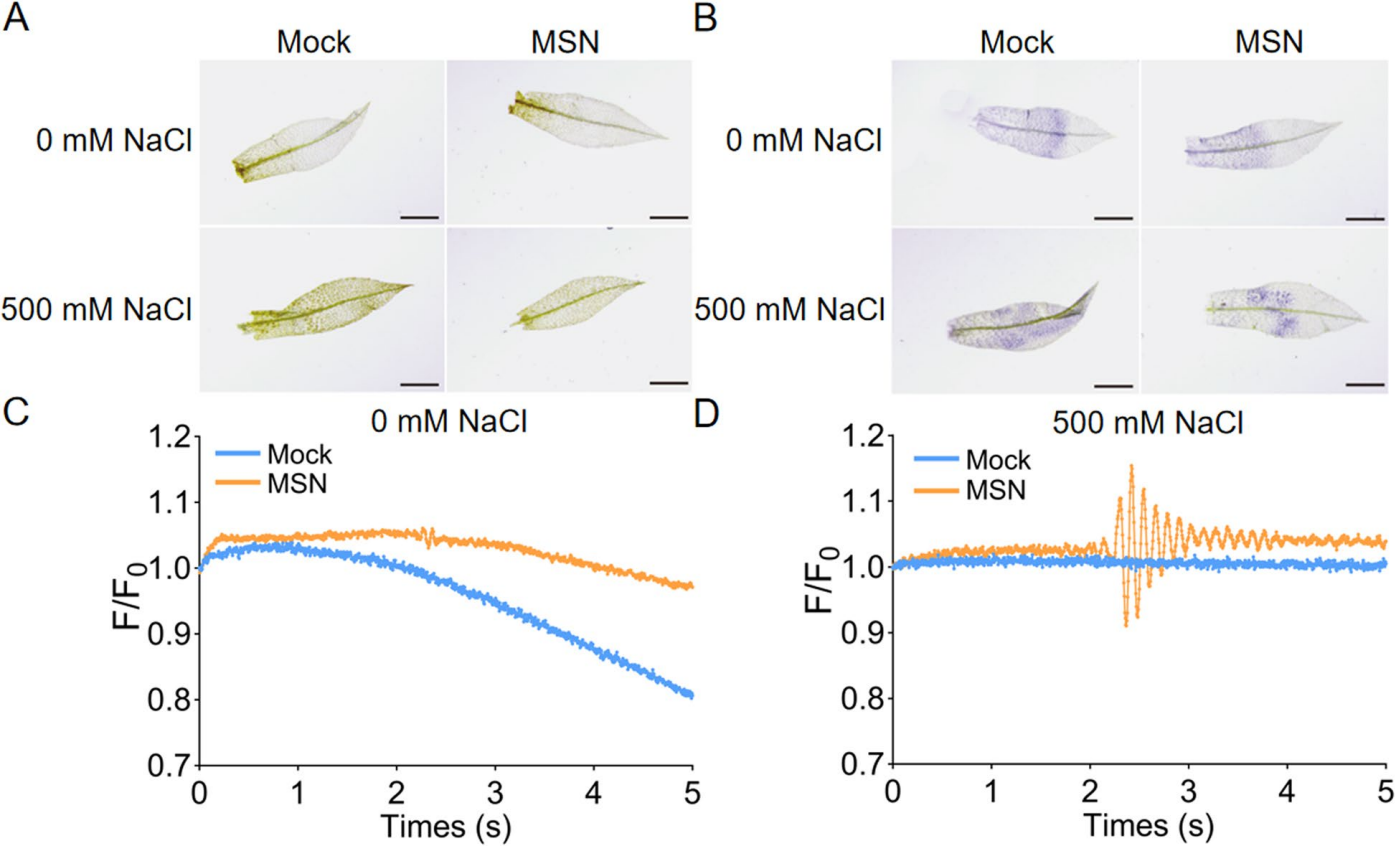
Effect of MSNs on ROS accumulation and subcellular Ca^2+^ oscillations in *P. patens* under NaCl stress. **A** Representative images of *P. patens* leaf staining with 3, 3′-diaminobenzidine (DAB) to detect hydrogen peroxide accumulation after 6 days of 500 mM NaCl stress, with or without 300 mg/L MSN treatment. **B** Representative images of *P. patens* leaf staining with nitro blue tetrazolium (NBT) to detect superoxide anion accumulation. Scale bar = 1 mm. **C** Ca^2+^ transients of *P. patens* treated with 300 mg/L MSN. Tips excised from one-month-old *P. patens* gametophores were soaked in 300 mg/L MSNs for 4 h and washed using sterile water, with sterile water serving as the non-treated mock group.** D** Ca^2+^ transients of *P. patens* treated with 300 mg/L MSN and under 500 mM NaCl. F_0_ represents the initial fluorescence intensity. F represents the real-time fluorescence intensity

To investigate the role of MSN in enhancing salt stress tolerance in *P. patens*, we employed 3,3′-diaminobenzidine (DAB) and nitro blue tetrazolium (NBT) staining to assess changes in hydrogen peroxide (H_2_O_2_) and superoxide anion (O_2_·^−^) accumulation (Fig. 4A and B). In the absence of salt stress, MSN treatment did not significantly affect ROS accumulation, as indicated by staining patterns similar to the mock condition, suggesting that MSN itself does not induce ROS accumulation significantly. However, when plants were subjected to 500 mM NaCl stress, both DAB and NBT staining revealed a substantial increase in ROS levels in the leaves, as evidenced by darker staining. Notably, the leaves treated with MSN exhibited reduced staining compared to untreated plants, indicating a reduction in ROS accumulation (Fig. 4A and B). This suggests that while NaCl induced substantial accumulation of H_2_O_2_ and O_2_·^−^, MSN treatment effectively mitigated ROS buildup in the leaves of *P. patens*.

Calcium ions (Ca^2+^) are essential intracellular signaling molecules, and their concentration changes directly reflect cellular responses of plants to NaCl stress. To investigate whether MSN influence Ca^2+^-mediated signaling in *P. patens* under salt stress, real-time subcellular Ca^2+^ oscillations in *P. patens* were recorded using Ca^2+^ transient measurement (Fig. 4C and D). The MSN treatment exhibited slightly elevated and sustained fluorescence intensity compared to the Mock treatment, which showed a stable and gradual decline in fluorescence intensity, suggesting an enhancement of basal calcium signaling by MSN (Fig. 4C). Under NaCl treatment, the MSN-pretreated plants displayed more pronounced and dynamic changes in fluorescence intensity, characterized by a clear oscillatory pattern and higher peak values, compared to MSN non-pretreated plants (Fig. 4D). This indicates an amplified calcium signaling response to salt stress when MSNs were applied. Overall, these results suggest that MSNs can reduce ROS accumulation and enhance the robustness of calcium signaling in *P. patens* under NaCl-induced stress, highlighting their potential role in modulating plant tolerance to salt stress.

### MSNs are distributed on the cell surface, partially enters cells and can be transported in vivo

To determine whether exogenously applied MSN can be internalized by the cells of *P. patens*, we conjugated MSN with a fluorescent marker (FITC) and observed its distribution using confocal microscopy (Figure S5). After 4 h of treatment with 9 mg/mL MSN-FITC, the FITC fluorescence signal displayed a flocculent distribution, primarily localized in the cytoplasm and potentially within vacuoles or vesicles. The FITC signal did not exhibit significant enrichment along cell contours, nor did it overlap with chloroplast autofluorescence. Furthermore, the signal did not present as dot-like or filamentous structures typically associated with mitochondria (Figure S5).

**Fig. 5:**
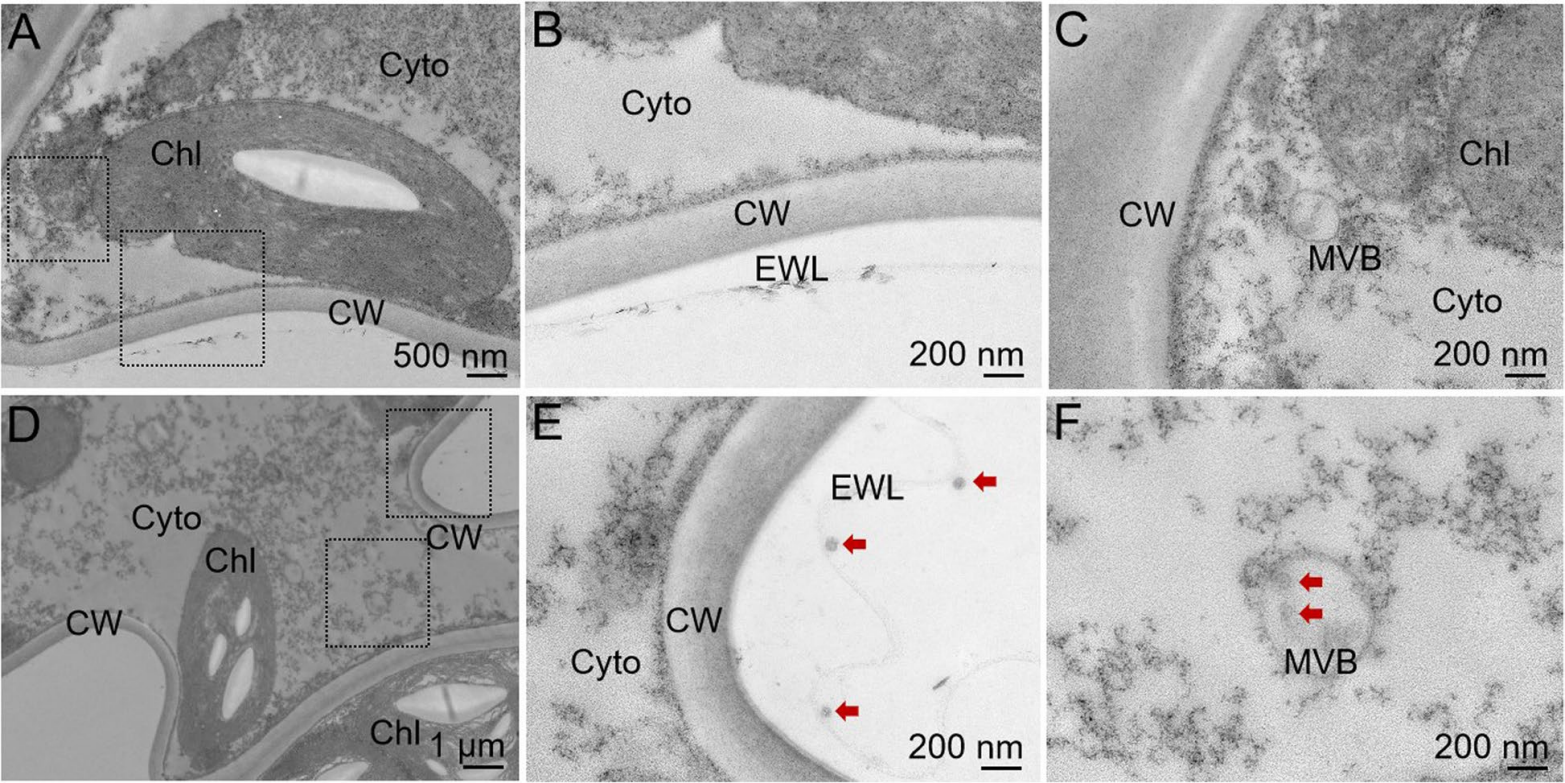
TEM observation of MSN distribution in *P. patens* leaves. **A**, **B** and **C** MSNs were not observed anywhere in the mock group. **D**, **E** and **F** MSNs were observed in the cuticular wax layer outside the cell wall, cytoplasm and vesicles. CW: Cell wall, Cyto: Cytoplasm, EWL: Epicuticular wax layer, MVB: Multivesicular body. The red arrows indicate MSNs. Scale bars are provided in the figure

To further confirm the localization of MSN nanoparticles, we treated *P. patens* leaves with 300 mg/L MSN for 4 h and examined the samples using TEM (Fig. 5 and Figure S6). TEM results revealed the presence of MSNs in the epicuticular wax layer, the cytoplasm, and vesicular structures. No MSNs were observed in the untreated plants (Fig. 5A and C). In treated plants, MSNs were not detected in the nuclei or chloroplasts (Fig. 5D and F, Figure S6). These findings indicate that MSNs primarily accumulate in the epicuticular wax layer of *P. patens* leaves, and a smaller fraction of nanoparticles is internalized into cells, where they are mainly localized in cytoplasmic vesicles.

**Fig. 6:**
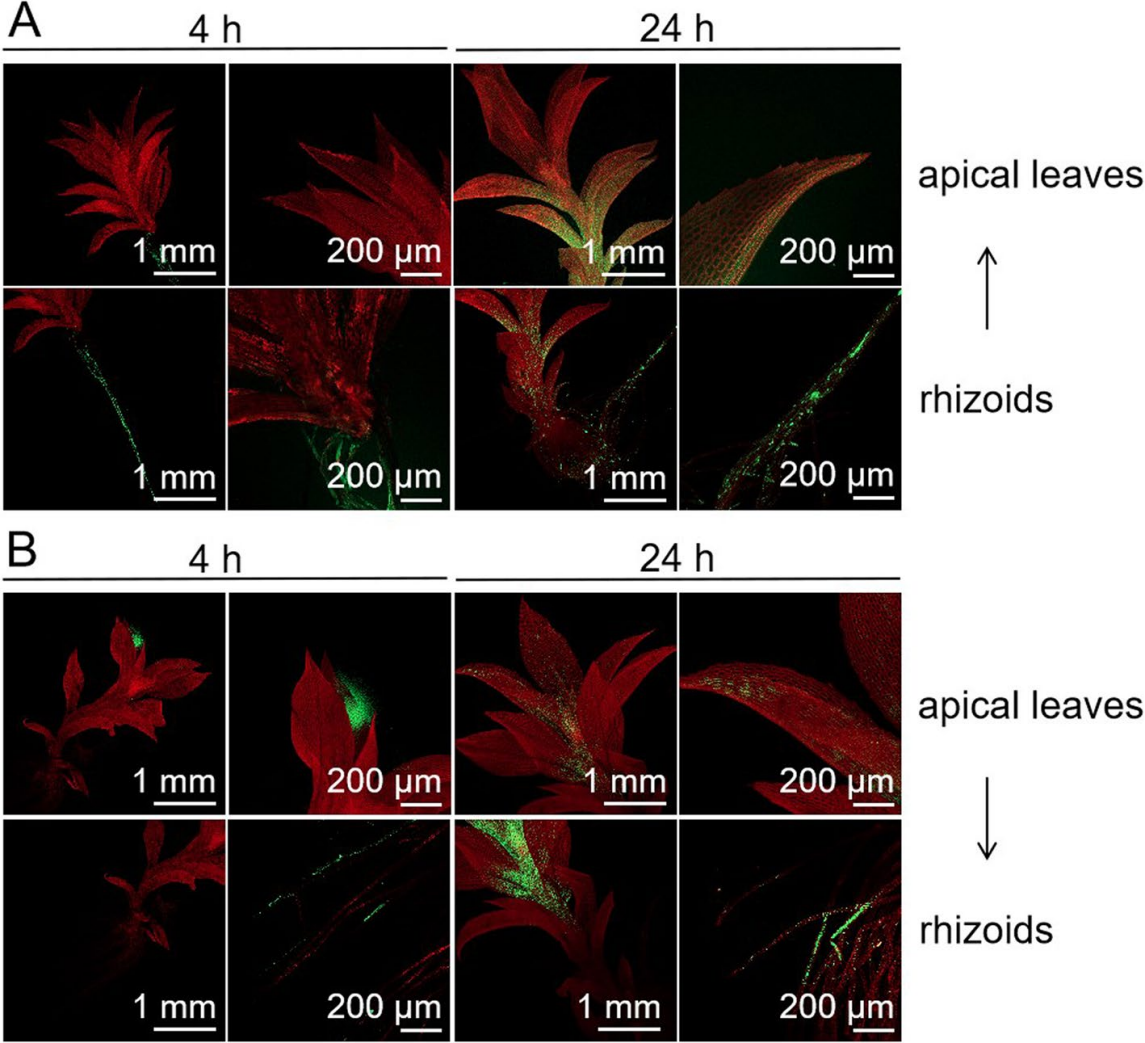
Intercellular transport and tissue distribution of MSN-FITC in *P. patens* gametophores observed under confocal laser scanning microscopy. **A** Rhizoids treated with 9 mg/mL MSN-FITC and fluorescence observed in rhizoids, stems, and apical leaves after 4 and 24 h. **B** Apical leaves treated with 9 mg/mL MSN-FITC and fluorescence observed in rhizoids, stems and apical leaves after 4 and 24 h. Scale bars are indicated in each subfigure

To investigate whether MSNs can be transported between cells and tissues within *P. patens* gametophores, we treated either the rhizoids (basal structures of the gametophore) or the apical leaves with 9 mg/mL MSN-FITC and observed the distribution of FITC fluorescence using confocal laser scanning microscopy after 4 and 24 h of exposure (Fig. 6). At 4 h, the FITC fluorescence signals were primarily localized in the treated tissue, either the rhizoids or apical leaves. However, after 24 h, the gametophores exhibited fluorescence signals in multiple tissues, including untreated regions. Specifically, when rhizoids were treated with MSN-FITC, fluorescence signals were observed in the stem and apical leaves at 24 h (Fig. 6A). Conversely, when apical leaves were treated, fluorescence signals were detected in the stem and rhizoids after 24 h (Fig. 6B). Taken together, these results indicate that MSNs can be transported intracellularly and intercellularly across different tissues of *P. patens*.

### Transcriptional regulation of stress-responsive genes in *P. patens* by MSN under NaCl treatment

To investigate the transcriptional changes induced by MSN under NaCl stress *in P. patens*, a comparative transcriptomic analysis was performed. Detailed metrics summarizing clean reads, including clean reads pairs, clean bases, read lengths, quality scores (Q20 and Q30), and GC content, were provided in Table S2. The sequencing data exhibited excellent quality, with Q20 and Q30 values exceeding 98% and 95%, respectively. Following filtering, the high-quality base pairs exceeded 6 × 10^9^ bps. Additionally, the Pearson correlation heatmap of samples showed strong correlations (Figure S7A), confirming high reproducibility among biological replicates and the reliability of the transcriptomic data.

To identify DEGs modulated by MSN under NaCl stress, thresholds of |log_2_ (Fold Change)| > 1.5 and False Discovery Rate (FDR) < 0.05 were applied. The volcano plot revealed a total of 1008 DEGs, of which 455 were upregulated and 553 were downregulated in MSN-treated plants compared to non-treated plants under 500 mM NaCl stress (Figure S7B and Table S3). GO enrichment analysis indicates that the DEGs were primarily involved in “response to stimulus”, “metabolic process”, “binding activity”, and “transporter activity” (Figure S7C). KEGG pathway enrichment analysis further highlighted significant pathways including “membrane transport”, “signal transduction”, “energy metabolism”, and “environmental adaptation” (Figure S7D). These findings suggest that MSN modulates transcriptional responses in *P. patens* under salt stress by promoting pathways involved in stress signaling, oxidative stress mitigation, and metabolic regulation, ultimately enhancing salt stress tolerance.

**Fig. 7:**
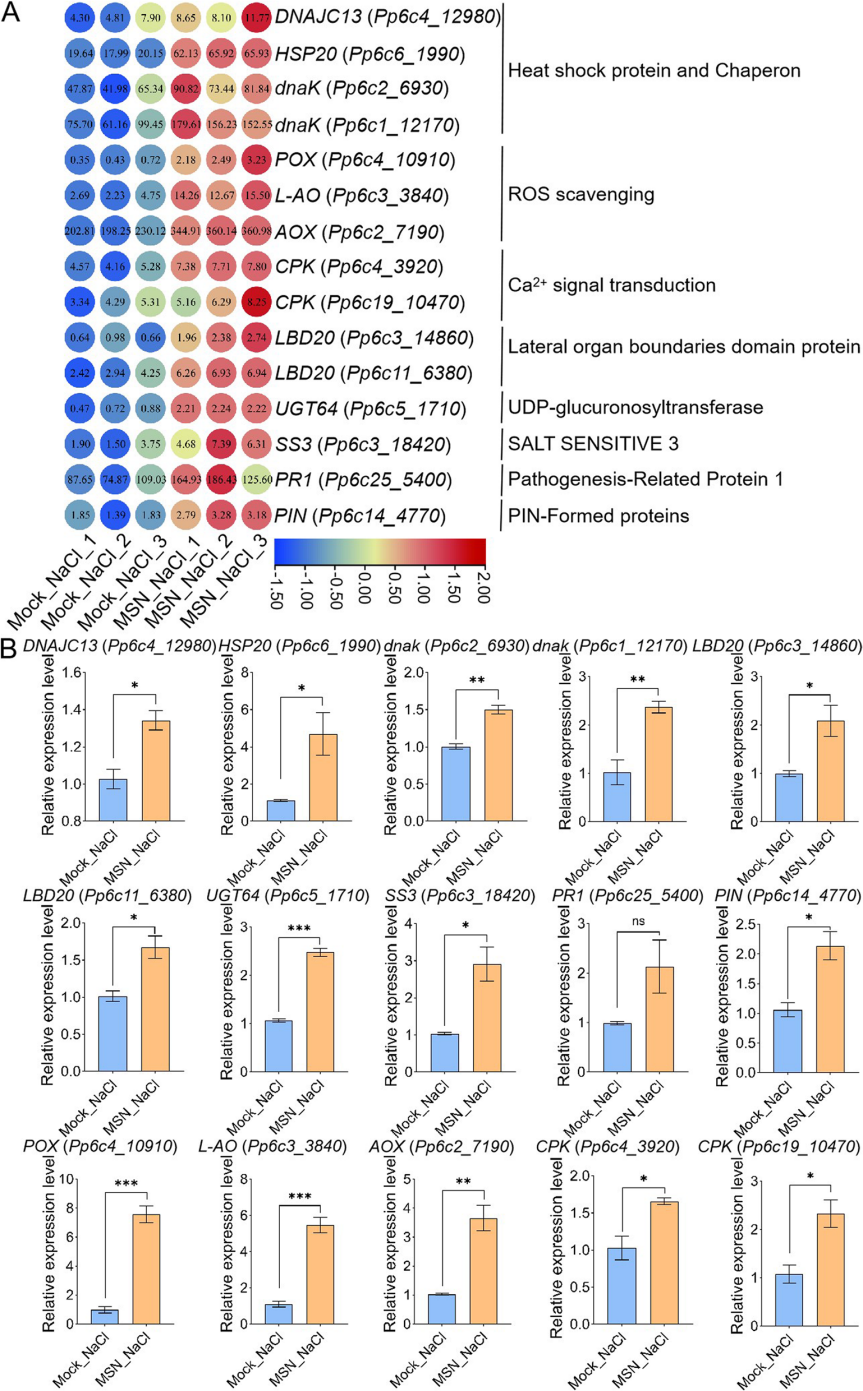
Differential expression analysis of *P. patens* gametophyte clusters following MSN treatment. **A** The heatmaps of *P. patens* on 500 mM NaCl at 300 mg/L MSN. These genes are derived from differentially expressed genes (DEGs) related with abiotic stress (salt stress). The FPKM (fragments per kilobase per million) values are shown in each circle. Using row scale and the scale method is normalized. Gene identifier shown in brackets after each protein name. **B** The qRT-PCR of the genes related with abiotic stress (salt stress). Data are presented as means ± SEM (*n* = 3). Asterisks indicate the significant difference between Mock_NaCl and MSN_NaCl. ns indicates no significant difference, * *p* < 0.05, ** *p* < 0.01, *** *p* < 0.001 (*t-test*)

To further examine the role of specific stress-responsive genes, key genes were selected based on their functions in abiotic stress response. A heatmap (Fig. 7A) showed that genes involved in ROS scavenging, such as *peroxidase* (*POX*), *L-ascorbate oxidase* (*L-AO*) and *alternative oxidase* (*AOX*), were significantly upregulated in MSN-treated plants under NaCl stress, highlighting the enhanced antioxidant capacity provided by MSN. Similarly, genes encoding heat shock proteins and chaperones, including *dnaJ heat shock protein family (HSP40) member C13* (*DNAJC13*) and *heat shock protein 20* (*HSP20*), showed increased expression, indicating improved protein stability and folding by MSN under salt stress conditions. The calcium signaling-related gene *calcium-dependent protein kinase* (*CPK*) was also upregulated in MSN-treated plants, suggesting amplified calcium-mediated signaling, which is crucial for stress signal transduction. Additionally, developmental and metabolic genes, such as lateral organ boundaries domain protein (*LBD20*), *PIN-Formed proteins* (*PIN*), *UDP-glucuronosyltransferase* (*UGT*) and *SALT SENSITIVE3* (*SS3*), displayed elevated expression, reflecting enhanced metabolic adjustments and developmental regulation in the presence of MSN. Stress marker genes, such as *PR1*, were also significantly upregulated under salt stress with MSN treatment, indicating enhanced defense responses.

To validate the transcriptomic results, qRT-PCR analysis was performed on selected DEGs (Fig. 7B). Under salt stress conditions, expression levels of *POX* (*Pp6c4_10910*), *L-AO* (*Pp6c3_3840*), *AOX* (*Pp6c2_7190*) and *CPK* (*Pp6c4_3920* and *Pp6c19_10470*) were 7.56, 5.46, 3.66, 1.65 and 2.33 times higher, respectively, in MSN treated plants compared to non-treated plants. Similar results were observed for *DNAJC13* (*Pp6c4_12980*), *HSP20* (*Pp6c6_1990*), *dnak* (*Pp6c2_6930* and *Pp6c1_12170*), *LBD20* (*Pp6c3_14860* and *Pp6c11_6380*), *UGT64* (*Pp6c5_1710*), *SS3* (*Pp6c3_18420*), *PR1* (*Pp6c25_5400*) and *PIN* (*Pp6c14_4770*), further supporting the reliability of the transcriptomic data. These results demonstrate that MSN enhances salt stress tolerance in *P. patens* by modulating key stress-responsive pathways, including ROS scavenging, calcium signaling, and metabolic regulation. These findings provide insights into the potential of MSN as an effective tool for improving plant resilience to abiotic stress.

## Discussion

In this study, we explored the effects of two silica-based nanomaterials, MSNs and VMSNs, on the model moss species *P. patens*. We observed that MSNs were distributed on the cell surface of *P. patens*, with some nanoparticles entering the cytoplasm and vesicles (Fig. 5). This is consistent with previous studies where nanoparticles, including MnONP and ZnO, were found to accumulate on the cell wall even after washing (Ghosh et al. 2019; Motyka et al. 2019b). In higher plant species, SiNPs have also been shown to accumulate on plant surfaces, forming a physical barrier that may protect plants from stress (Merwad et al. 2018; Li et al. 2023). Our findings further revealed that MSNs not only accumulated in the cuticle wax layer outside the cell wall but also penetrated cells and were transported within the gametophyte tissues (Figure S6, Figs. 5 and 6). While previous studies in other plant species, such as wheat, Arabidopsis, and rice, suggest that nanoparticles may enter plant cells through stomata, hydrophilic pores, or by directly damaging the cuticle (Nadiminti et al. 2013; Larue et al. 2014; Avellan et al. 2019; El-Shetehy et al. 2021), *P. patens* lacks stomata in its gametophyte leaves (Caine et al. 2016), leaving the exact mechanism of MSN entry into moss cells unclear. Nevertheless, similar phenomena have been observed in other studies. For instance, nanoparticles such as nano-TiO_2_ and ZnO showed increased uptake with exposure time and successfully penetrated moss tissue (Ghosh et al. 2019). Additionally, Fe nanoparticles penetrated the leaves of the bryophyte *Aphanorrhegma* when applied as mineral water suspensions (Canivet et al. 2014). Our study also indicates that nanoparticles successfully entered moss cells, similar to previous reports. Interestingly, the method of external application of nanomaterials seems to influence their penetration into cells. In our experiments, as in others where intracellular nanoparticles were detected, the immersion method was employed. When Ag-Nano was incorporated into the culture medium, despite its high toxicity to *P. patens*, TEM images showed no nanoparticles entered the cells. This suggests that nanoparticle entry may depend on the method of external application, though the exact mechanism remains to be further investigated.

Existing research on nanoparticles in mosses has primarily focused on metal nanoparticles such as nano-ZnO, MnONP and AgNPs, which negatively affect moss growth and stress resistance through oxidative stress, genotoxicity, and epigenotoxicity (Liang et al. 2018; Ghosh et al. 2019; Motyka et al. 2019a). For instance, AgNPs were found to be toxic to the growth of *P. patens* gametophytes, with toxicity closely related to the concentration and surface coatings (Liang et al. 2018). In contrast, the nanoparticles used in this study are silica nanoparticles, which are derived from silicon, a trace element that plays an essential role in plant growth, development, and stress responses (Coskun et al. 2019). Studies have demonstrated that silicon improves growth and stress tolerance in crops such as rice, cucumber, sorghum, tomato, soybean (Artyszak 2018; Gou et al. 2020; Zhu et al. 2020; Ismail et al. 2022). Silicon application has been shown to reduce oxidative stress, alleviate damage, and regulate plant hormones like abscisic acid and gibberellin to improve seed germination (Shi et al. 2014; Gou et al. 2020). Nano-silica application in agriculture have also gained attention, with findings showing enhance plant stress tolerance by increasing antioxidant activity, and reducing reactive oxygen species (Farhangi-Abriz and Torabian 2018). Additionally, silica nanoparticles have been shown to promote rice root development, improve drought resistance, and enhance wheat growth by increasing auxin and fructose content (Du et al. 2022; Li et al. 2023). In this context, our study provides evidence that silica nanoparticles are non-toxic to mosses and can improve their tolerance to salt stress. Moreover, the differential effects of MSN and VMSN on moss salt resistance, likely due to their distinct surface morphologies, highlight the complexity of nanoparticles-moss interactions. Other studies have shown that factors such as size and concentration affect their effectiveness too (Yusefi-Tanha et al. 2022).

Bryophytes are pioneer plants that thrive in environments where higher plants struggle. Enhancing their salt tolerance could expand their ecological distribution, enabling them to thrive in diverse ecosystems, accelerate soil desalination, improve soil quality, and create favorable conditions for other plants. Mosses exhibit exceptional adaptability to stress due to their unique photosynthetic processes, water absorption and retention capabilities, and ability to regulate metabolic activities (Rensing et al. 2008, 2020; Khraiwesh et al. 2015). For instance, Antarctic moss has been shown to withstand high salt stress, with the ROS scavenging system playing a critical role in this resilience (Zhang et al. 2019). However, nanoparticles sometimes induce oxidative stress in mosses. For example, nano-ZnO reduces L-ascorbic acid (AsA) concentrations significantly (Motyka et al. 2019a). In contrast, MSN treatment in this study effectively reduced the accumulation of H_2_O_2_ and O_2_·^−^ under salt stress, thereby enhancing the survival rate of *P. patens* (Fig. 4). This is consistent with studies on bio-selenium nanoparticles (bioSeNPs), which significantly reduced DAB and NBT staining in salt-stressed seedlings (El-Badri et al. 2022). Transcriptome analyses, including GO and KEGG enrichment, along with qRT-PCR validation (Fig. 7 and Figure S7), further revealed that under salt stress, MSN treatment upregulated genes involved in ROS scavenging, such as *POX*, *L-AO* and *AOX*, thereby enhancing antioxidant capacity in *P. patens*. Chloroplasts and the process of photosynthesis are crucial physiological aspects affected by salinity stress (Vineeth et al. 2023). The moss gene, *PaPC*, can enhances photosynthesis and salt stress tolerance in Arabidopsis without affecting growth (No et al. 2025). Our study showed that MSN treatment significantly improved the Fv/Fm ratio and survival rates of *P. patens* under salt stress (Figs. 2 and 3), providing further evidence of its protective effects. Calcium signaling also plays a pivotal role in maintaining cell membrane integrity, regulating ion transport, and activating adaptive responses (Qudeimat et al. 2008). In this study, MSN treatment slightly increased baseline calcium signaling under normal conditions, with a significant enhanced signal under salt stress (Fig. 4). The upregulation of *CPK* in MSN-treated plants further links molecular responses to observed physiological changes (Fig. 7). This suggests that MSN treatment enhances stress perception and activate downstream adaptive responses. Finally, MSN treatment upregulated genes involved in developmental and metabolic processes, such as *LBD20*, *PIN*, *UGT64* and *SS3*, as well as chaperones like *DNAJC13* and *HSP20*, which are critical for protein stability and proper folding (Fig. 7). These results further support enhanced metabolic and protein functional adjustments of MSN treatment under salt stress. Therefore, the improved Fv/Fm ratios and survival rates observed in MSN treated mosses can be attributed to the modulation of key molecular pathways including oxidative stress mitigation, calcium signaling, protein stability, and metabolic regulation (Scheme 1).

## Conclusion

Mosses play a vital role in ecosystem functions, including soil formation and improvement, air purification, climate regulation, biodiversity maintenance, and environmental monitoring. Additionally, mosses possess significant economic value, such as medicinal and ornamental uses. Enhancing the salt tolerance of mosses offers the potential to expand their ecological distribution and cultivation range, improve their yield and quality, and create new opportunities for the utilization of moss resources. This study revealed that silica-based nanoparticle MSNs are non-toxic to moss growth. MSNs were observed to localize on the plant surface, with some nanoparticles entering plant cells and being transported across tissues. Notably, MSN treatment significantly improved moss resistance to salt stress. Physiological measurements demonstrated that increased survival rates were associated with enhanced Fv/Fm ratios, modulation of calcium-regulated signaling pathways, and reduced oxidative damage caused by ROS accumulation. On a molecular level, transcriptome analysis and gene expression validation confirmed that MSN treatment modulates key pathways involved in oxidative stress mitigation, calcium signaling, protein stability, and metabolic regulation. These findings provide valuable insights into the potential of nanomaterials in plant science, particularly in enhancing the ecological tolerance and resilience of mosses in challenging environments.

## Materials and methods

### Chemicals

Cetyltrimethylammonium Bromide (CTAB), Tetraethyl orthosilicate (TEOS), and 3-aminopropyltriethoxysilane (APTES) were purchased from MACKLIN (Shanghai, China). Fluorescein isothiocyanate (FITC) and N-Hexadecyltrimethylammonium Chloride (CTAC) were obtained from Aladdin (Shanghai, China). Trolamine (TEA), cyclohexane, acetone, ethanol and sodium hydroxide (NaOH) were sourced from SINOPHARM (Beijing, China).

### Synthesis of VMSN via a two-phase diffusion system

VMSNs were synthesized using a two-phase diffusion system. For the aqueous phase, 1 g of CTAB and 0.8 mL NaOH (0.1 M) were dissolved in 50 mL of deionized water and maintained at 60°C. For the organic phase, 20 mL of TEOS was mixed with 80 mL of cyclohexane to form a homogeneous solution at a volume ratio of 20% (v/v). The mixture was then added dropwise to the aqueous phase under continuous stirring, forming a two-phase reaction system with the organic phase layered on top of the aqueous phase.

The two-phase reaction system was maintained at 60°C with constant stirring for 72 h to ensure complete reaction and formation of the VMSN structure. After the reaction, the mixture was collected by centrifugation at 11,000 rpm, yielding VMSN@CTAB. The product was washed with deionized water and ethanol sequentially. To remove CTAB template, VMSN@CTAB were redispersed in 50 mL of acetone and refluxed at 50°C for 12 h. The purified VMSNs were then collected and stored for further use.

### Synthesis of MSN via alkaline catalysis hydrolysis method

MSNs were synthesized using an alkaline-catalyzed hydrolysis method. Initially, 20 g of CTAC was dissolved in 200 mL of deionized water and stirred at 95°C until fully dissolved. Subsequently, 600 mg of TEA was added to the solution and stirred for 1 h until the mixture became completely transparent. Then, 15 mL of TEOS was added into the reaction system and maintained at 95°C for an additional hour to facilitate the formation of the MSN@CTAC composite. The product, MSN@CTAC, were collected by centrifugation and washed three times with deionized water and methanol to remove residual reactants.

Subsequently, 0.5 g of the synthesized MSN@CTAC were redispersed in 80 mL of methanol, followed by the addition of 4.5 mL of concentrated hydrochloric acid. The mixture was refluxed at 60°C for 48 h to remove the CTAC template. The MSNs were then obtained by centrifugation and washed sequentially with deionized water and methanol before being stored for further use.

### Synthesis of MSN-FITC

Fluorescent label FITC was conjugated to the surface of MSN via a condensation reaction involving amino and carboxyl groups. To prepare MSN-NH_2_, 1 g of MSN was dispersed homogeneously in 100 mL of ethanol and heated to 70°C with stirring. APTES (2 mL) was then added dropwise to the suspension, and the mixture was refluxed at 70°C for 24 h in a condensation apparatus. The product was collected by centrifugation at 11,000 rpm for 25 min, followed by sequential washing with water and ethanol to yield MSN-NH_2_.

To attach FITC, 20 mg of MSN-NH_2_ was dispersed in 10 mL of ethanol, and 1 mg of FITC was added to the suspension under dark condition. The reaction was allowed to proceed for 24 h in the dark. The resulting MSN-FITC was collected by centrifugation (11,000 rpm, 25 min). The quantity of FITC conjugated to the MSN was determined by measuring the fluorescence intensity.

### Characterization of MSNs and VMSNs

The morphology of MSNs and VMSNs was examined using transmission electron microscopy (TEM, TECNAI G2 20 S-TWIN), confirming the successful synthesis of two distinct nano-silica formulations. Particle size and dispersity were measured and assessed using dynamic light scattering (DLS, Malvern Nano-ZS90). Successful labeling of FITC molecules on MSN was verified using an ultraviolet–visible (UV–Vis) spectrophotometer (Alpha-1860plus). Further characterization, including the efficiency of FITC labeling, dispersibility, and drug-loading potential of the silica particle formulation, was evaluated using a fluorescence spectrophotometer (Shimadzu RF-6000) and a small animal in vivo imaging system (Spectral AMIX).

### Plant growth conditions and treatments

The moss *P. patens* Gransden 2004 ecotype, provided by Prof. Mitsuyasu Hasebe, was used as the wild type (WT) in this study. Plants were grown on BCD medium supplemented with 1 mM CaCl_2_ at 25°C under a 16 h light/8 h dark photoperiod with a light intensity of 60–80 μmol photons m^−2^ s^−1^. For salt stress assays, 2–3 mm tips excised from one-month-old *P. patens* gametophores were transferred to BCD medium containing different concentrations of NaCl (0 mM, 50 mM, 150 mM, 300 mM, 500 mM and 800 mM), with 0 mM NaCl serving as the control. Photos were taken at 5, 10, and 15 days.

For MSN and VMSN treatments, various concentrations of MSN and VMSN, ranging from 0.1 mg/L to 300 mg/L (specifically, 0.1 mg/L, 1 mg/L, 10 mg/L, 50 mg/L, 100 mg/L and 300 mg/L), were dissolved in sterile water to assess the effect of *P. patens* to nano-silica formulations under salt treatments. For the control group, an equivalent volume of sterile water was used. The 2–3 mm tips from 1-month-old gametophores were immersed in different concentrations of MSN or VMSN for a duration of 4 h. Subsequently, they were rinsed thoroughly with sterile water and placed onto the BCD medium supplemented with 500 mM NaCl to induce salt stress for further evaluation.

### Measurement of the growth area, survival rate, and maximum quantum yield of photosystem II (Fv/Fm)

The plants were pretreated with nanoparticles for 4 h followed by being placed on BCD containing 500 mM NaCl for 6 days. The representative images were obtained using Nikon camera. The Fv/Fm was photographed and measured by a pulse-amplitude modulation fluorometer (Heinz Walz GmbH, HEXAGON-IMAING-PAM) and a FluorCam fluorescence imaging systema (Photon Systems Instruments, FluorCam 800MF). Plants for measurement at stress stage and recovery stage were kept in the dark for 30 min before measurement. After the 6-days salt stress, plants were transferred to BCD medium for a 60-days recovery period. The growth area was measured by Image J. The survival rate was calculated as the ratio of the number of viable gametophyte to the number of total gametophyte placed per dish in the BCD medium.

### Nitro blue tetrazolium (NBT) and 3,3′-diaminobenzidine (DAB) staining

Plants were pretreated with nanoparticles for 4 h and them placed on BCD medium containing 500 mM NaCl for 6 days. For NBT staining, leaves were incubated with 1 mg/mL of NBT (Shanghai yuanye Bio-Technology, S19048) in potassium phosphate buffer (pH 7.8) for the detection of superoxide anion radicals. For DAB staining, leaves were incubated with 1 mg/mL of DAB (Shanghai yuanye Bio-Technology, S19134) in potassium phosphate buffer (pH 3.8) for 2 h to visualize hydrogen peroxide. After staining, the samples were decolorized in 95% ethanol, and images were taken using a stereo microscope (Olympus corporation, SZX2-ILLTQ).

### The mitochondrial Ca^2+^uptake assay in *P. patens*

The fluorescent dye Rhod-2/AM (Thermo Fisher Scientific, R1244) was used to measure mitochondrial Ca^2+^ uptake, following the manufacturer’s instructions (Li et al. 2022). Briefly, the 2–3 mm tips from nanoparticle-treated or non-treated 1-month-old gametophore were incubated with 3 mg/L Rhod-2/AM by vacuum pump for 30 min to allow the cells to load the dyes. After incubation at 37 °C for 45 min and rinsed with sterile water to remove the excessive dye, the samples were observed on optical mapping system (SciMedia, MiCAM02LEX2-LZ4) according to the manufacturer’s instructions.

### Transmission electron microscopy (TEM) for ultrastructural analysis

For transmission electron microscopy, one-month-old single gametophores were soaked in 300 mg/L MSN for 4 h, followed by rinsing with sterile water. The leaves were transferred to fresh TEM fixation buffer (Servicebio, G1102) and cut into 1–2 mm^3^ pieces, as previously described by Du et al. (Du et al. 2022). Ultrastructural imaging was performed using a transmission electron microscopy (Hitachi, HT7800).

### Fluorescence microscopy of MSN-FITC

The rhizoids and apical leaves of one-month-old single gametophores were exposed to 9 mg/mL MSN-FITC for 2 s to observe the uptake of MSN-FITC. After exposure, the samples were incubated at room temperature for 4–24 h. After washed with sterile water for 3 times, the fluorescence signals were observed using confocal microscopy (ZEISS, LSM 980). Excitation was performed at 488 nm, and the emission filters for MSN-FITC were set to 520–541 nm.

### Isolation of plant RNA and Real-Time Quantitative PCR (qRT-PCR) analysis

Total RNA was extracted using TransZol (TransGen Biotech, Q30704) according to the manufacturer’s instructions. cDNA synthesis was conducted using MonScript™ RTIII All-in-One Mix with dsDNase (Monad, MR05101M). qRT-PCR was performed using ArtiCan^CEO^ SYBR qPCR Mix (Beijing Tsingke Biotech, TSE401) on a Bio-Rad CFX384 system. The primer sequences utilized for qRT-PCR are listed in Supplementary Table S1.

### RNA Sequencing (RNA-seq) and transcriptome analysis

One-month-old gametophores were subjected to 500 mM NaCl salt stress for 10 days with or without 300 mg/L MSN treatment. The samples were immediately transferred to liquid nitrogen. Total RNA is extracted using HiPure Universal RNA Mini Kit (Magen, R4130-02). The quality of extracted RNA is assessed using Nanodrop 2000 and Agilent 2100 to determine RNA integrity. mRNA was enriched using Oligo (dT) magnetic beads, purified, and used to construct a sequencing library according to the manufacturer’s instructions with the VAHTS Universal V6 RNA-seq Library Kit for MGI (VAzyme, Nanjing, China). The prepared libraries were sequenced using an MGI high-throughput sequencer (Yuenyong et al. 2018). Raw sequencing data were processed using SOAPnuke (v2.1.0) for quality filtering. Quality control included evaluating read quality, base composition, and adapter contamination. Low-quality bases and adapters were trimmed, and reads were filtered to remove any potential contaminants or low-complexity sequences. Differential gene expression analysis was performed by fitting models to estimate gene expression levels and dispersion. Volcano plots were used to visualize the statistical significance (-log_10_FDR) versus log_2_fold change in gene expression between treatment and control groups. Differential expressed genes (DEGs) were identified using the thresholds: |log_2_ (Fold Change)| > 1.5 and False Discovery Rate (FDR) < 0.05. Gene Ontology (GO) enrichment analysis is performed to annotate DEGs and classify them into biological processes, molecular functions, and cellular components. Kyoto Encyclopedia of Genes and Genomes (KEGG) pathway analysis is conducted by KOBAS (V3.0) to elucidate the metabolic pathways and interactions involved in the observed gene expression changes. Pathway enrichment analysis was performed using *R* software with self-written scripts and BH correction. The raw sequencing data have been submitted to the BioProject database with the accession ID PRJNA1184830.

### Illustration and statistical analysis

Scheme 1 was created with BioGDP.com. In this study, all experiments were performed with three biological replicates. Student’s *t*-*test* was used for hypothesis testing in statistics between two samples by GraphPad Prism version 8.0. Statistical analysis for group comparisons was conducted using *Two-way* *ANOVA*, followed by post-hoc *Tukey tests*. Significant differences in figures were indicated by asterisks *, ** and ***, corresponding to *p* < 0.05, *p* < 0.01 and *p* < 0.001, respectively.

## Supplementary Information

Supplementary Material 1: Figure S1. TEM observation of MSN, MSN-FITC and VMSN-FITC, and average size. ns indicates no significant difference (*two-way ANOVA*, *Tukey test*).

Supplementary Material 2: Figure S2.  Salt stress responses of *P. patens*.

Supplementary Material 3: Figure S3.  The effects of different concentrations of nanoparticales on *P. patens* under salt stress.

Supplementary Material 4: Figure S4. Growth area of *P. patens* during the recovery stage.

Supplementary Material 5: Figure S5. Localization of MSN-FITC in *P. patens* leaves observed under confocal laser scanning microscopy after 4 hours of treatment with 9 mg/mL MSN-FITC.

Supplementary Material 6: Figure S6. Localization of MSN in *P. patens* leaves observed by transmission electron microscopy after 4 hours of treatment with 300 mg/L MSN.

Supplementary Material 7: Figure S7. Transcriptome analysis of *P. patens* comparing MSN treatment vs Mock of under NaCl stress.

Supplementary Material 8: Table S1. The primers used for qRT-PCR.

Supplementary Material 9: Table S2. Clean reads information table following adapter sequence filtering of raw sequencing data in *P. patens *treated and ntreated with MSN under 500 mM sodium chloride conditions.

Supplementary Material 10: Table S3. The number of upregulated and downregulated genes in the volcano plot are 455 and 553 respectively, along with their specific names.

## Data Availability

The raw sequencing data have been submitted to the BioProject database with the accession ID PRJNA1184830. The materials used in this study will be made available to researchers upon reasonable request.

## Abbreviations

*P. patens*: *Physcomitrium patens*
MSNs: Mesoporous silica nanoparticles
VMSNs: Virus-like mesoporous silica nanoparticles
*CPK*: *Calcium-dependent protein kinase*
*LBD20*: *Lateral organ boundaries domain gene 20*
*PIN*: *PIN-formed protein*
*UGT64*: *UDP-glucuronosyltransferase 64*
*SS3*: *Salt sensitive3*
*PR1*: *Pathogenesis-related protein 1*
*dank*: *Dnak heat shock protein*
*DNAJC13*: *Dnaj heat shock protein family (Hsp40) member C13*
POX: Peroxidase
L-AO: L-ascorbate oxidase
AOX: Alternative oxidase
HSP40: Heat shock protein 40
HSP20: Heat shock protein 20
TEM: Transmission electron microscopy
DLS: Dynamic light scattering
FITC: Fluorescein isothiocyanate
CTAB: Cetyltrimethylammonium Bromide
TEOS: Tetraethyl orthosilicate
APTES: 3-Aminopropyltriethoxysilane
CTAC: N-Hexadecyltrimethylammonium Chloride
TEA: Trolamine
Fv/Fm: Maximum quantum yield of photosystem II
DAB: 3,3’-Diaminobenzidine
NBT: Nitro blue tetrazolium
qRT-PCR: Quantitative reverse transcription PCR
RNA-seq: RNA sequencing
UV–Vis: Ultraviolet–visible spectrophotometer
*ANOVA*: Analysis of variance
DEGs: Differentially expressed genes
FDR: False discovery rate
GO: Gene Ontology
KEGG: Kyoto Encyclopedia of Genes and Genomes
WT: Wild type
H_2_O_2_: Hydrogen peroxide
O_2_^·−^: Superoxide anion
ROS: Reactive oxygen species
SOD: Superoxide dismutase
CAT: Catalase
