# Supplementary figures and images for: Nontoxic mesoporous silica nanoparticles protect Physcomitrium patens against salt stress
Source: Stress Biol. 2025 Nov 14;5(1):69. doi: 10.1007/s44154-025-00262-5 (PMC12615889; doi:10.1007/s44154-025-00262-5)

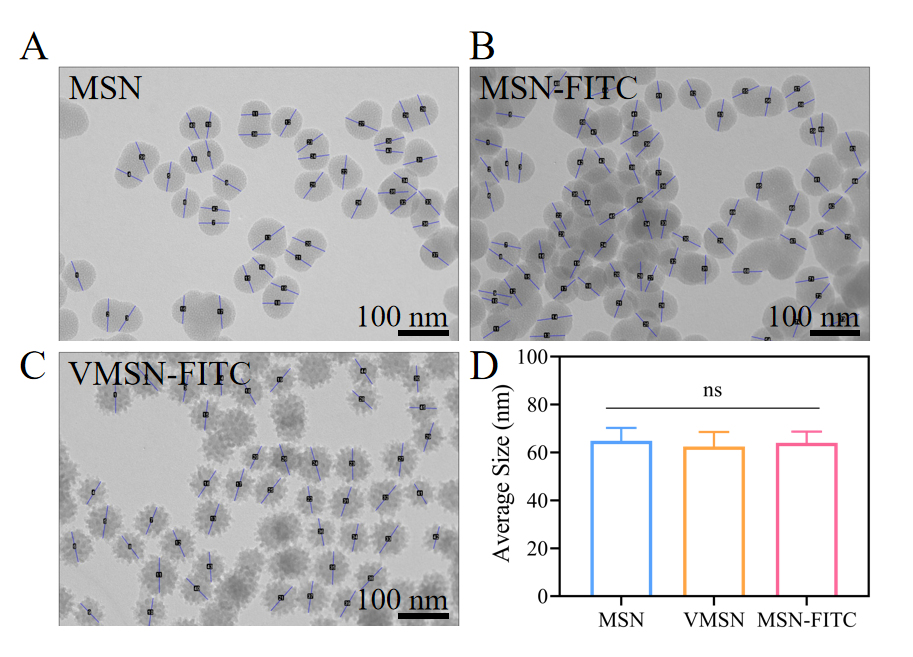

Supplement: Supplementary file 1 — Supplementary Material 1: Figure S1. TEM observation of MSN, MSN-FITC and VMSN-FITC, and average size. ns indicates no significant difference (two-way ANOVA, Tukey test). [file 44154_2025_262_MOESM1_ESM.jpg]

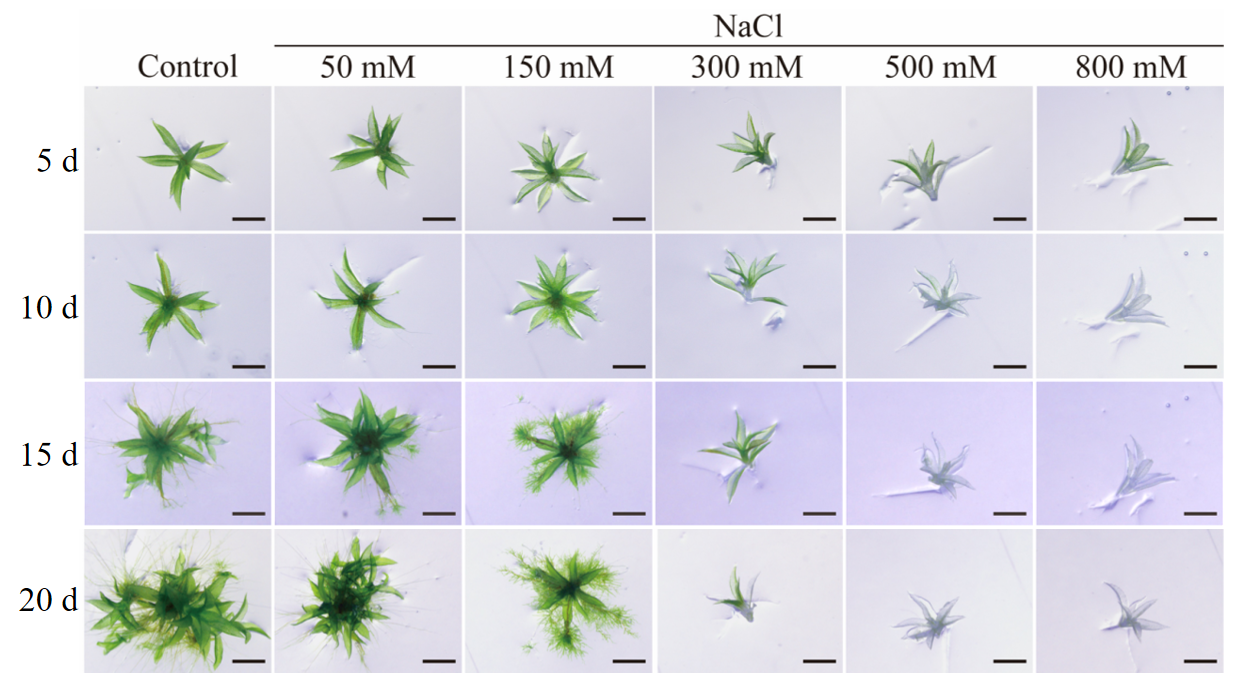

Supplement: Supplementary file 2 — Supplementary Material 2: Figure S2. Salt stress responses of P. patens. [file 44154_2025_262_MOESM2_ESM.jpg]

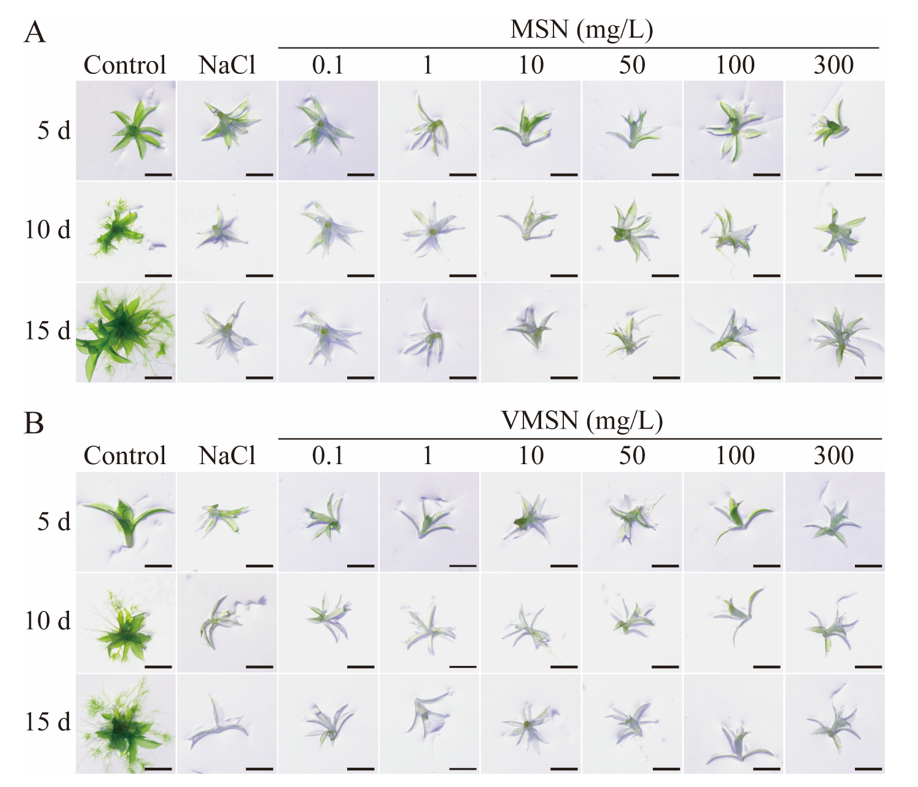

Supplement: Supplementary file 3 — Supplementary Material 3: Figure S3. The effects of different concentrations of nanoparticales on P. patens under salt stress. [file 44154_2025_262_MOESM3_ESM.jpg]

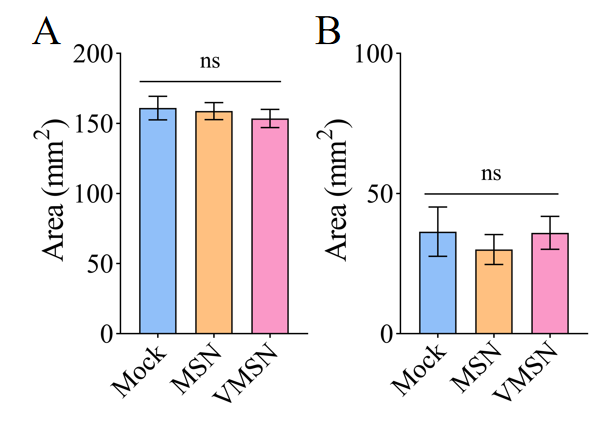

Supplement: Supplementary file 4 — Supplementary Material 4: Figure S4. Growth area of P. patens during the recovery stage. [file 44154_2025_262_MOESM4_ESM.jpg]

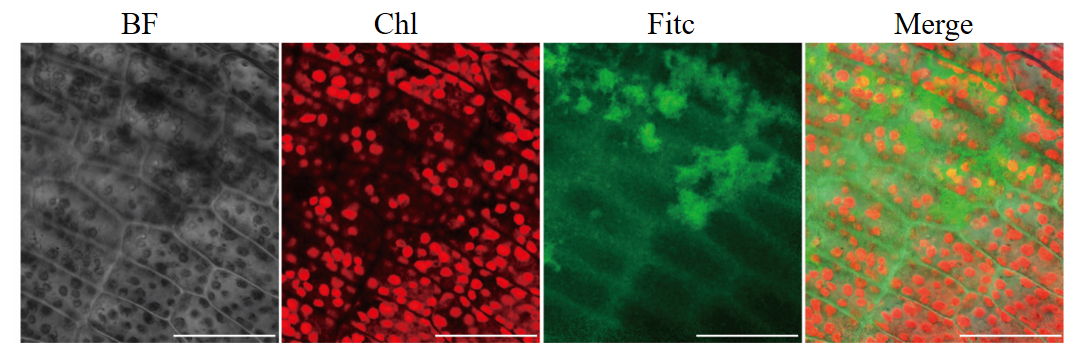

Supplement: Supplementary file 5 — Supplementary Material 5: Figure S5. Localization of MSN-FITC in P. patens leaves observed under confocal laser scanning microscopy after 4 hours of treatment with 9 mg/mL MSN-FITC. [file 44154_2025_262_MOESM5_ESM.jpg]

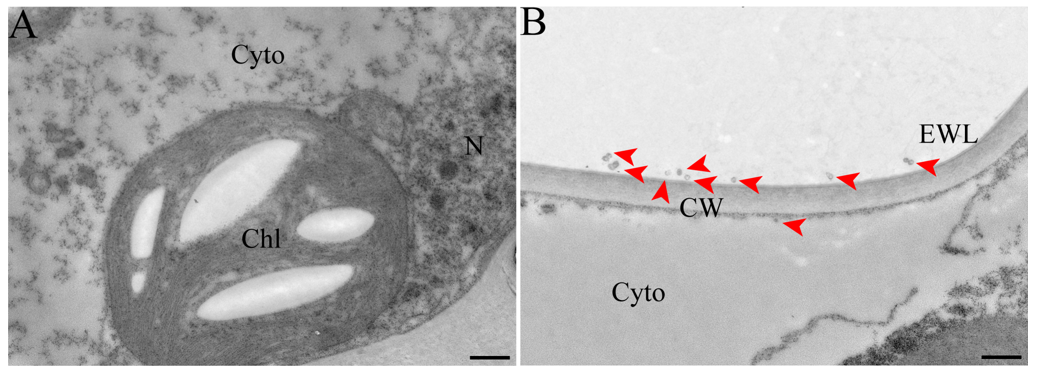

Supplement: Supplementary file 6 — Supplementary Material 6: Figure S6. Localization of MSN in P. patens leaves observed by transmission electron microscopy after 4 hours of treatment with 300 mg/L MSN. [file 44154_2025_262_MOESM6_ESM.jpg]

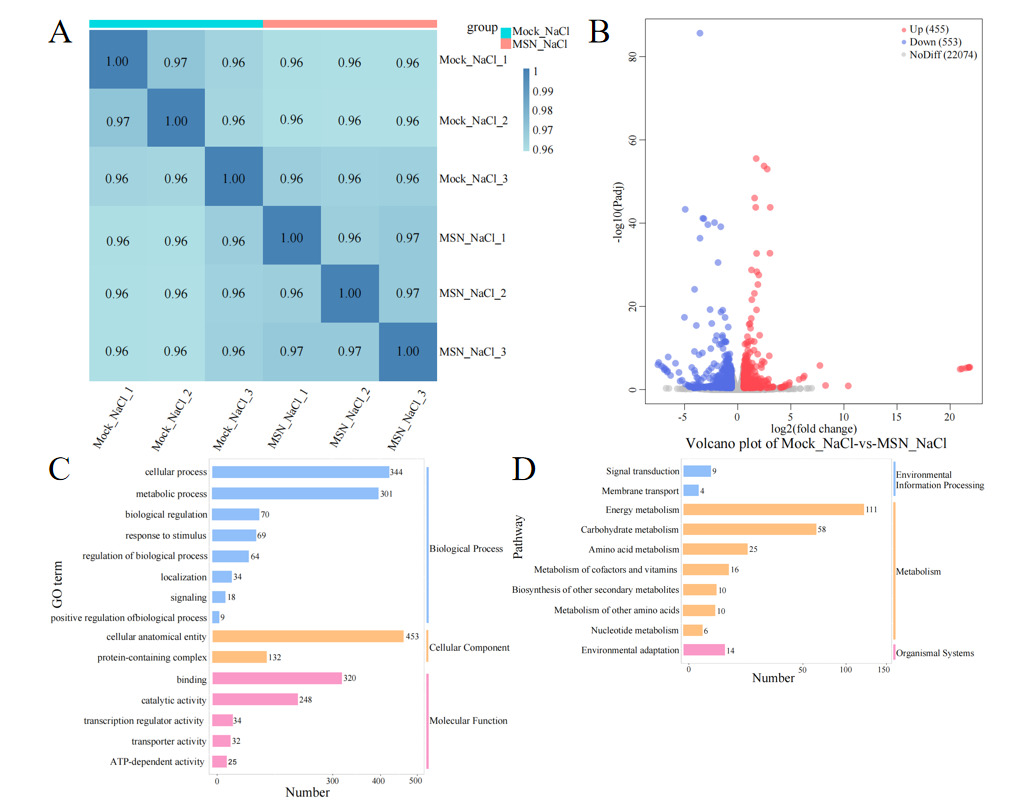

Supplement: Supplementary file 7 — Supplementary Material 7: Figure S7. Transcriptome analysis of P. patens comparing MSN treatment vs Mock of under NaCl stress. [file 44154_2025_262_MOESM7_ESM.jpg]
